# Exploring the role of curcumin, nanocurcumin, and a PPAR agonist in preventing paraquat-induced systemic inflammation and oxidative stress in rats

**DOI:** 10.22038/ijbms.2025.82057.17753

**Published:** 2025

**Authors:** Mahboobeh Ghasemzadeh Rahbardar, Mohammad Ehsan Taghavizadeh Yazdi, Sima Beigoli, Hamideh Amin, Mohammad Hossein Boskabady

**Affiliations:** 1 Applied Biomedical Research Center, Mashhad University of Medical Sciences, Mashhad, Iran; 2 Department of Physiology, School of Medicine, Mashhad University of Medical Sciences, Mashhad, Iran

**Keywords:** Curcumin, Inflammation, Oxidative stress, Paraquat, Pioglitazone, PPAR gamma

## Abstract

**Objective(s):**

We investigated the effects of curcumin, nanocurcumin, and pioglitazone, a peroxisome proliferator-activated receptor gamma (PPARγ) activator, on the systemic inflammation and oxidative stress induced by inhaled paraquat (PQ).

**Materials and Methods:**

The experimental design included male Wistar rats divided into nine groups. Animals of the control group (Ctrl) were exposed to saline and those of other groups to 54 mg/m^3^ PQ aerosols 8 times on alternate days. PQ exposing groups were treated with saline (PQ group), curcumin (30 mg/kg, Cu), nanocurcumin (2 and 8 mg/kg, NC-L, and NC-H), pioglitazone (5 mg/kg, Pio), Pio+ Cu-L, Pio + NC-L, and dexamethasone (0.03 mg/kg, Dexa). Pio was administered intraperitoneally and other treating agents by gavage for 16 days during the PQ exposure period. Total and differential white blood cell (WBC) counts, malondialdehyde (MDA), superoxide dismutase (SOD), catalase (CAT), thiol, interleukin (IL)-10, and tumor necrosis factor-alpha (TNF-α) levels were measured.

**Results:**

The inhalation of PQ increased total WBC, differential WBC, MDA, IL-10, and TNF-α blood levels. It also decreased blood levels of CAT, SOD, and thiol. The treatment groups (Cu, NC-L, NC-H, Pio+Cu, Pio+NC-L, Pio, and Dexa) ameliorated PQ-induced alterations. Furthermore, the improvements in most parameters in the Pio+Cu and NC-L-treated group were more significant than the results of the three substances individually.

**Conclusion:**

The amelioration of systemic inflammation and oxidative stress caused by inhaled PQ by Cu, NC, and Pio were shown. Furthermore, the findings indicated a synergistic effect between Pio with Cu and NC, suggesting the involvement of PPARγ-mediated mechanisms in the effects of curcumin.

## Introduction

The herbicide paraquat (PQ), which is extensively utilized, has been associated with adverse effects on human health since it can cause inflammation and systemic oxidative stress (1). Exposure to PQ has been linked to various toxicological effects, including lung injury (2), renal dysfunction (3), and neurotoxicity (4). The underlying mechanism of PQ-induced systemic oxidative stress and inflammation involves triggering lipid peroxidation and lowering thiol content, and suppressing the activities of superoxide dismutase (SOD) and catalase (CAT), leading to cellular damage and activation of the inflammatory response (5, 6). Additionally, the administration of PQ has been demonstrated to trigger lung (7) and systemic inflammation (5, 8) through the elevation of tumor necrosis factor-alpha (TNF-α), interleukin (IL)-1β, and IL-6 (7). Inhalation of PQ has been associated with increased total and differential white blood cell (WBC) counts, indicating an inflammatory response (6, 8).

While several medicines, such as anti-oxidants and anti-inflammatory agents, have been used to mitigate the effects of PQ toxicity (9), their efficacy is often limited, and they may possess undesirable side effects. Therefore, an urgent need is to explore alternative therapeutic strategies and identify novel compounds that can effectively alleviate PQ-induced systemic inflammation and oxidative stress, promoting better outcomes for affected individuals. Research projects are paying increasing attention to the possible advantages of herbal medicines as alternative therapies for illness prevention as antitoxic agents (10-14). In recent decades, the therapeutic potential of medicinal plants, such as *Ginkgo biloba* (15), *Zataria multiflora* (5, 16, 17), *Crocus sativus *(18), quercetin (19), and naringenin (20), has been highlighted in the management of PQ-induced toxicity.

Turmeric, scientifically known as *Curcuma longa*, belongs to the *Zingiberaceae* family and is a perennial herbaceous plant with rhizomes. It is native to regions of southeast Asia and India. Turmeric roots have long been used as a popular spice in cooking, bringing taste and color to various recipes. Turmeric contains several bioactive compounds, with curcumin (Cu) being the primary component responsible for its therapeutic properties (21). Modern physio-pharmacological studies revealed the remarkable properties of turmeric and Cu, including anti-oxidant, anti-inflammatory (21, 22), anticancer (23), immunomodulatory (22), antirheumatic (24), and hypnotic (25), antidote (8, 26, 27). However, one major limitation of Cu is its poor bioavailability, which impedes its oral therapeutic efficacy (28). To overcome this challenge, researchers have turned to nanotechnology, developing nanocurcumin (NC) formulations that enhance its solubility and absorption in the body. NC has emerged as a promising approach to improve the pharmacological advantages of Cu and expand its application in medicinal studies (29). By harnessing the potential of nanotechnology, researchers aim to unlock the full therapeutic potential of Cu, paving the way for more effective treatments and interventions.

In our previous research, the treatment effects of NC in oxidative stress, inflammation, and lung tissue pathology induced by PQ aerosol were examined in which NC rats were exposed to PQ during days 1–15 and received treatments on days 16–31. However, the current study aims to evaluate the preventive potential of Cu, NC, pioglitazone (Pio), a peroxisome proliferator-activated receptor gamma (PPARγ) activator, and dexamethasone on PQ-induced systemic inflammation and oxidative stress in rats. Rats were exposed to PQ inhalation from days 1 to 15 and concurrently received treatments. By evaluating key parameters such as total and differential white blood cell counts, inflammatory markers (TNF-α and IL-10), and oxidative stress markers (MDA, SOD, CAT, and thiol), this research attempts to shed light on the preventive efficacy of these compounds and their role in ameliorating the deleterious effects of paraquat exposure.

## Materials and Methods

### Chemicals

PQ and dexamethasone were obtained from Sigma-Aldrich Chemical Co., St. Louis, MO, USA. Pio was acquired from Samisaz Pharmaceutical Company, Iran. Ethanol 96% was purchased from Betagene laboratory equipment, Mashhad, Iran. Curcumin was sourced from Sami-Sabinsa Group in Bangalore, Karnataka, India.

### Preparation of NC

The process involves the following steps:

1. Initial preparation: 100 mg of Cu is added to the oily phase. The mixture is stirred using a magnetic stirrer at 500 rpm for two hours at room temperature.

2. Sonication: After the stirring step, the mixture is placed in a sonicator bath for one hour. This process helps to achieve a clear, yellow, homogeneous oily solution.

3. Nanoemulsion formation: Deionized water is added to the oily phase at a ratio of 5:1 (w/w). The mixture is then stirred at 500 rpm for 30 min, forming the final nanoemulsion.

4. Characterization: The prepared NC particles exhibit spherical shapes with a uniform size distribution. The mean diameter of the NC particles, determined using transmission electron microscopy (TEM) results and analyzed with Image Tools and SPSS software, is reported to be 15.7 ± 3.55 nm.

5. Stability and solubility: The prepared NC is shown to be stable for at least three months and soluble in water (8, 30).

### Animals

Male Wistar rats, aged 8–9 weeks, were obtained from the animal house of the School of Medicine, Mashhad University of Medical Sciences (MUMS), Mashhad, Iran. These rats weighed approximately 230 ± 34 g on average. The rats were individually housed in steel cages under controlled conditions, including a 12-hour light/dark cycle and a temperature of 22 ± 2 °C, with a relative air humidity of 54 ± 2%. Throughout the experiment, the rats had free access to water and food. All experimental procedures followed the guidelines and regulations provided by the ethical committee of MUMS for Animal Experiments (ID: 961810). 

### Study protocol

Sixty-three healthy male Wistar rats were assigned to 9 study groups (n=7):

1. Control group: Rats were exposed to saline aerosol every other day (8 times, each time for 30 min) for 16 days.

2. PQ groups: Rats were exposed to PQ aerosol every other day (8 times, each time for 30 min). Animals of PQ-exposed groups were treated during PQ exposure (16 days) with the following agents.

2.1. Saline as PQ group

2.2. Cu (30 mg/kg) as Cu group (31)

2.3. NC (2 mg/kg) as NC-L group (2)

2.4. NC (8 mg/kg) as NC-H group (2)

2.5. Pio (5 mg/kg, IP) as Pio group (2)

2.6. Cu and Pio as Pio (8) + Cu group

2.7. NC-L and Pio as Pio (2) + NC-L group

**Figure 1:**
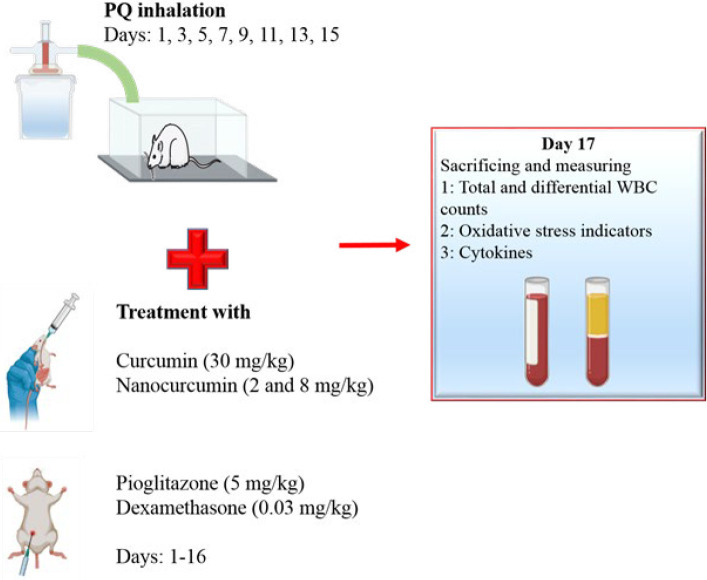
Experimental design depicting the administration of curcumin, nanocurcumin, and pioglitazone treatments to male Wistar rats exposed to paraquat (PQ) aerosols for evaluating systemic inflammation and oxidative stress

2.8. Dexamethasone (Dexa, 0.03 mg/kg) as Dexa group (8), (Figure 1).

Pioglitazone was administered intraperitoneally, and other agents were administered by gavage. It is essential to note that saline was used as the solvent to dissolve PQ. An Omron CX3 nebulizer from Japan, with a particle size ranging from 3 to 5 μm, was employed to generate PQ aerosol. The nebulizer operated at an airflow rate of 8 l/min. The aerosol produced was then directed into an exposure box, following a method described in a previous study (16). In the exposure box, a PQ dose of 54 mg/m^3^ was achieved (5, 32). Moreover, the doses, routes of administration, and administration period were selected according to a similar study (2, 8, 31).

### Preparation of blood samples, analysis of total and differential WBC counts, and biochemical parameters

On day 17 of the research, following the completion of the treatment period, the animals were anesthetized with an intraperitoneal injection of ketamine (50 mg/kg) and xylazine (5 mg/kg). Peripheral blood samples (5 ml) were obtained from the hearts of the animals. Four ml of the blood samples were then centrifuged for 10 min at 2000 rpm to separate the serum, which was then kept at -70 °C until further investigation of oxidative stress indicators and cytokines.

The differential WBC count was performed by creating a blood smear stained with Wright-Giemsa following the procedures described in previous research. The total WBC count was carried out in duplicate using a Neubauer chamber (33, 34). 

MDA, SOD, CAT, and thiol were evaluated using earlier established procedures (6). In summary, one ml of serum was mixed with two milliliter of thiobarbituric acid (TBA)/trichloroacetic acid (TCA)/HCl reagent, heated in a water bath for 40 min, cooled, then centrifuged at 1,000 ×g for 10 min in order to quantify MDA. The absorbance was determined at 535 nm, and the formula for calculating the MDA concentration (C) in nM was C = Absorbance/(1.56×105) (34).

The tetrazolium dye, MTT (3-(4, 5-dimethylthiazol-2-yl, 2, 5-diphenyltetrazolium bromide)) was reduced to its formazan by superoxide-dependent reduction, and this process was used to measure the activity of SOD. The formazan was measured at 570 nm and expressed as unit (U)/ml (34).

By measuring the decrease in absorbance at 240 nm/min and calculating the rate constant, k (dimension: s-1, k), of hydrogen peroxide breakdown, the activity of CAT was calculated (35) and given as unit (U)/ml (34).

The blood cytokine levels of IL-10 and TNF-α were determined using enzyme-linked immunosorbent assay (ELISA) kits from Karmania Pars, Kerman, Iran. The measurements were carried out per the manufacturer’s and previously published methods. To summarize, five ml of blood was obtained into a red vacuum tube and centrifuged for 10 min at 600 rpm. The serum was isolated and stored at -80 °C in cryovials until analysis. Using the cytokine detection kits, an ELISA was conducted to determine the levels of TNF-α and IL-10 in serum. As directed by the manufacturer, all reagent preparation, working standards, and protocols were followed. An ELISA reader (BIO-RAD) with dual filters set to 450 and 570 nm was used to measure the absorbance. Every sample was defrosted just once, and each sample was tested twice (36). Cytokine levels were measured in six rats from each group at random.

### Statistical analysis

Data normality was assessed using the Kolmogorov-Smirnov test. A one-way ANOVA test was performed to analyze biochemical and morphological data, followed by Tukey’s test for multiple comparisons. The results were presented as means ± SEM, following the convention of previous similar studies (8). A significance level of *P*<0.05 was considered statistically significant. All statistical analyses were conducted using the GraphPad Prism 8 software program (GraphPad Software, Inc., San Diego, CA, USA).

## Results

### Total and differential WBC

**Figure 2:**
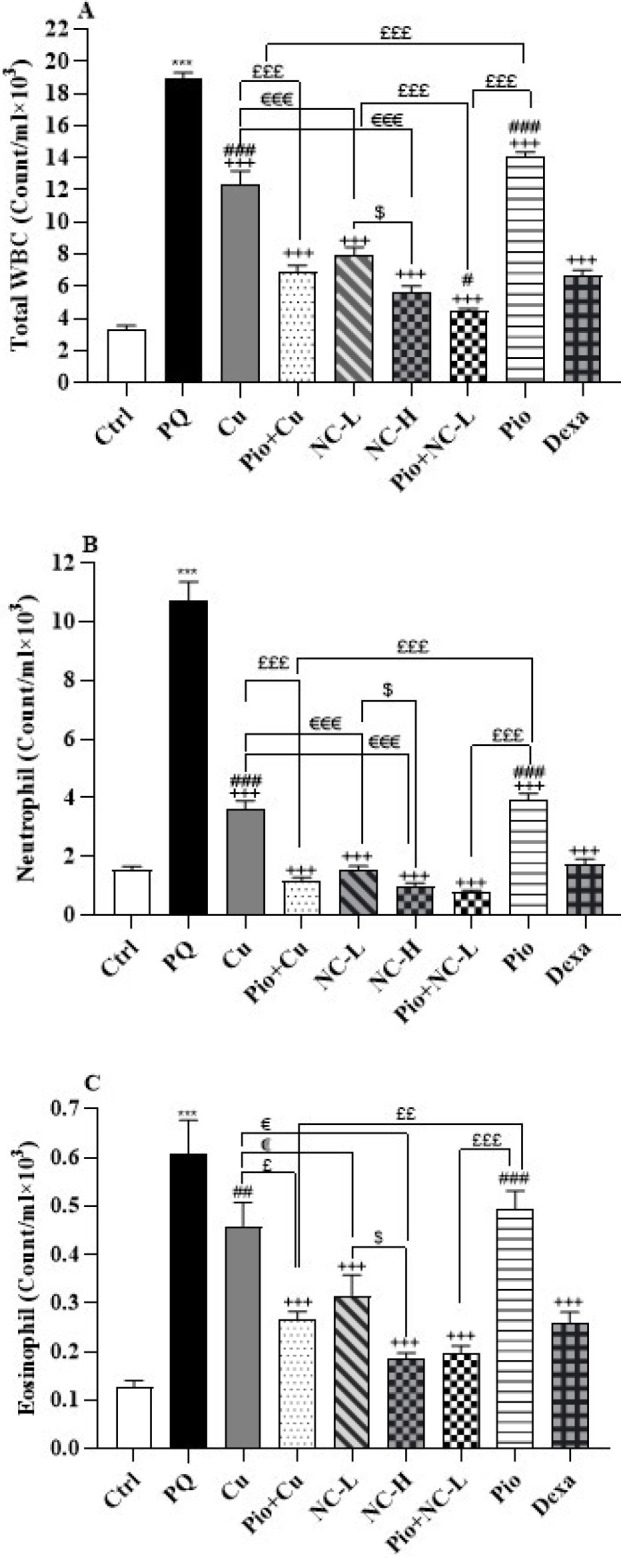
A: Total WBC, B: neutrophil, and C: eosinophil counts in the male Wistar rats' blood of the control group (Ctrl), group exposed to 54 mg/m^3 ^paraquat aerosol (PQ), exposing groups to PQ and treated with curcumin (30 mg/kg, 16 days, gavage) (Cu), nanocurcumin (2 mg/kg, 16 days, gavage) (NC-L), nanocurcumin (8 mg/kg, 16 days, gavage) (NC-H), Cu and pioglitazone (5 mg/kg, 16 days, IP) (Pio+ Cu), Pio+ NC-L, Pio, and dexamethasone (0.03 mg/kg, 16 days, IP) (Dexa)

**Figure 3:**
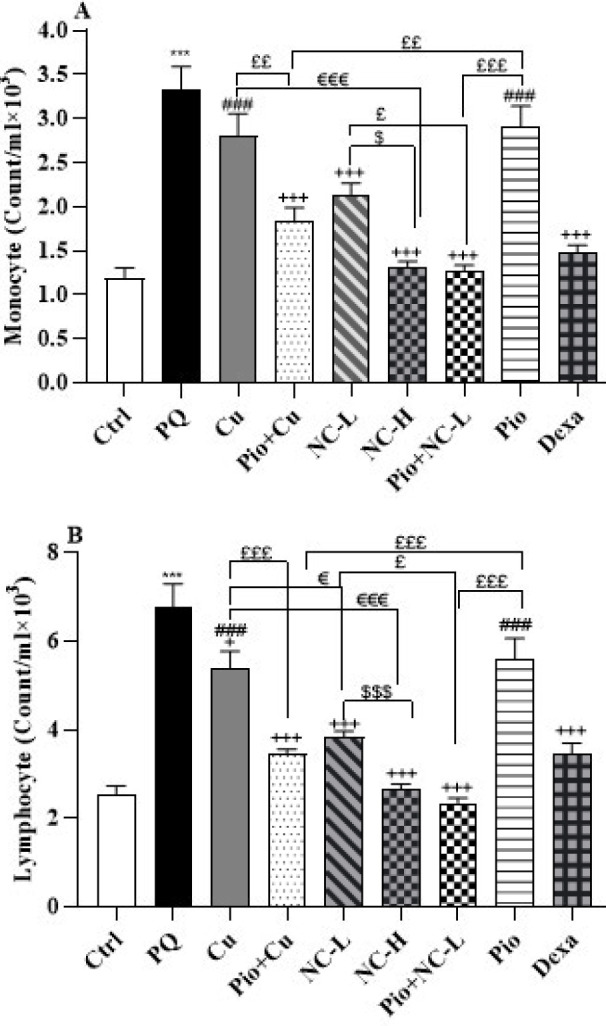
A: Monocyte and B: Lymphocyte counts in the male Wistar rats' blood (Counts/ml) of the control group (Ctrl), the group exposed to 54 mg/m^3^ paraquat aerosol (PQ), exposing groups to PQ and treated with curcumin (30 mg/kg, 16 days, gavage) (Cu), nanocurcumin (2 mg/kg, 16 days, gavage) (NC-L), nanocurcumin (8 mg/kg, 16 days, gavage) (NC-H), Cu and pioglitazone (5 mg/kg, 16 days, IP) (Pio+ Cu), Pio+ NC-L, Pio, and dexamethasone (0.03 mg/kg, 16 days, IP) (Dexa)

The obtained data revealed that PQ increased total WBC and differential WBC (neutrophils, eosinophils, monocytes, and lymphocytes) in comparison with the control group (*P*<0.001 for all) (Figures 2 and 3). 

Compared to the PQ group, the concurrent administration of PQ with Cu, Pio+ Cu, NC-L, NC-H, Pio + NC-L, Pio, and Dexa significantly reduced the numbers of total WBC and neutrophils (*P*<0.001). Furthermore, Cu (*P*<0.01), Pio+ Cu, NC-L, NC-H, Pio+ NC-L, Pio, and Dexa groups decreased the numbers of eosinophils (*P*<0.001). The data also illustrated that Pio+ Cu, NC-L, NC-H, Pio+ NC-L, and Dexa significantly lessened the numbers of monocytes (*P*<0.001) (Figure 3A). Besides, Cu (*P*<0.05), Pio+ Cu, NC-L, NC-H, Pio+ NC-L, and Dexa (*P*<0.001) groups reversed the increased lymphocytes numbers compared to the PQ group. 

NC-H group reduced the numbers of total WBC, neutrophils, eosinophils, monocytes (*P*<0.05 for all), and lymphocytes (*P*<0.001) more than the NC-L group.

In Group NC-L numbers of total WBC, neutrophils (*P*<0.001), and eosinophils (*P*<0.05) lowered more than in the Cu group. Moreover, in NC-L groups the numbers of lymphocytes lowered more than in the Cu group (*P*<0.05) (Figure 3).

In the Pio+Cu group, total WBC, neutrophils, and lymphocytes declined more than in the Pio and Cu groups (*P*<0.001 for all). The same results were obtained for the numbers of total WBC for Pio+NC-L compared to NC-L and Pio group (*P*<0.001). In addition, Pio+NC-L group had decreased neutrophils and eosinophils compared to the Pio group (*P*<0.001). In the Pio+Cu group, the number of eosinophils decreased more than in the Pio group (*P*<0.01) and the Cu group (*P*<0.05). In addition, the Pio+Cu group showed dropped numbers of monocytes more than the Pio and Cu groups (*P*<0.01 for both). Pio+NC-L showed decreased monocytes and lymphocytes more than NC-L (*P*<0.05) and Pio groups (*P*<0.001) (Figure 3).

The Dexa group showed more significant decreases in total WBC, monocyte, and lymphocyte levels than the Cu and Pio groups (*P*<0.001 for both cases). Compared to the Dexa group, only the Pio+NC-L group could attenuate the amounts of total WBC significantly (*P*<0.05). Moreover, the Dexa administration showed lowered neutrophil levels compared to the Cu and Pio groups (*P*<0.001). Likewise, Dexa supplementation decreased the eosinophils levels compared to the Cu (*P*<0.01) and Pio (*P*<0.001) groups (Figure 3).

### Oxidative stress markers

**Figure 4:**
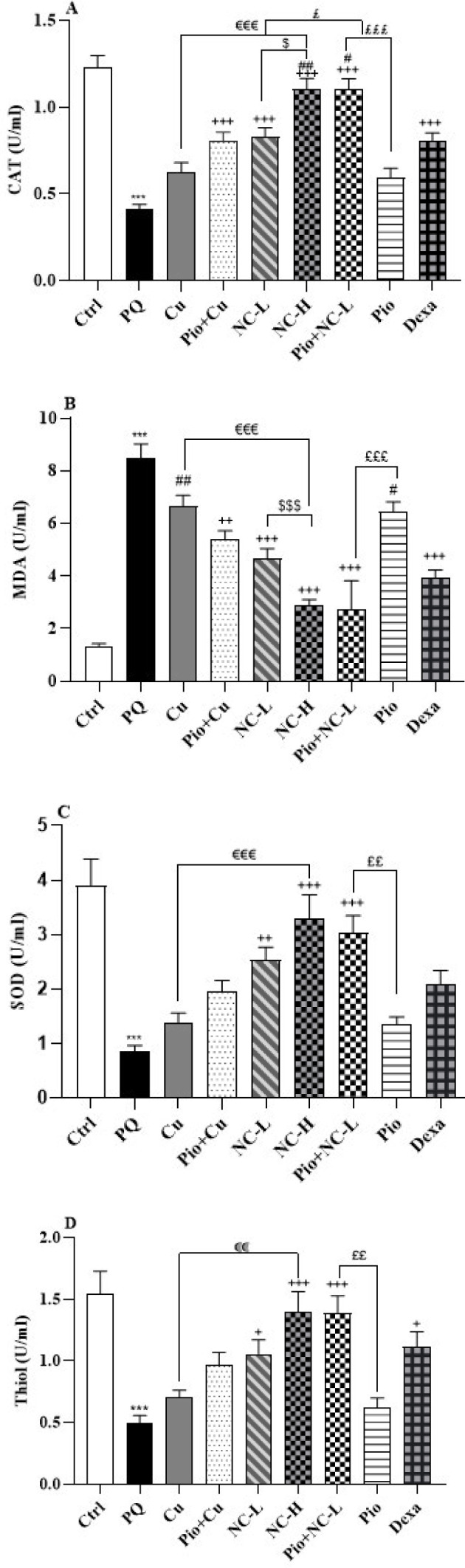
Oxidant and anti-oxidant biomarkers A: CAT, B: MDA, C: SOD, and D: thiol in the male Wistar rats' serum (Ctrl), group exposed to 54 mg/m^3^ paraquat aerosol (PQ), exposing groups to PQ and treated with curcumin (30 mg/kg, 16 days, gavage) (Cu), nanocurcumin (2 mg/kg, 16 days, gavage) (NC-L), nanocurcumin (8 mg/kg, 16 days, gavage) (NC-H), Cu and pioglitazone (5 mg/kg, 16 days, IP) (Pio+ Cu), Pio+ NC-L, Pio, and dexamethasone (0.03 mg/kg, 16 days, IP) (Dexa).

Exposure to PQ resulted in a significant decrease in the levels of CAT, SOD, and thiol, as well as an increase in the amounts of MDA (*P*<0.001 for all) (Figure 4). 

Pio+ Cu, NC-L, NC-H, Pio+ NC-L, and Dexa groups significantly boosted the levels of CAT (*P*<0.001). Moreover, Cu (*P*<0.01), NC-L, NC-H, Pio+ NC-L, and Dexa groups (*P*<0.001) attenuated the levels of MDA. As the results show, NC-L (*P*<0.01), NC-H, and Pio+NC-L (*P*<0.001) groups augmented SOD levels (Figure 4C). Similarly, NC-L (*P*<0.05), NC-H, and Pio+NC-L (*P*<0.001) groups could pointedly enhance thiol levels.

As shown in Figure 4A, the NC-H group showed increased CAT levels more than the NC-L group (*P*<0.05). Furthermore, the NC-H group had lower MDA levels than the NC-L group (*P*<0.001).

NC-H group showed increased CAT, SOD (*P*<0.001), and thiol (*P*<0.01) but reduced MDA levels more than Cu group (*P*<0.001).

Pio + NC-L group augmented CAT (*P*<0.001), SOD, and thiol (*P*<0.01) levels more than the Pio group. Also, the Pio+NC-L group had lessened levels of MDA compared to the Pio group (*P*<0.001) (Figure 4B).

In NC-L (*P*<0.001) and Pio + NC-L (*P*<0.05) groups, CAT levels increased more significantly than in the Dexa group (Figure 4A). Dexa group showed lowered MDA levels significantly more than Cu (*P*<0.01) and Pio (*P*<0.05) groups.

### Cytokine levels of IL-10 and TNF-α

**Figure 5:**
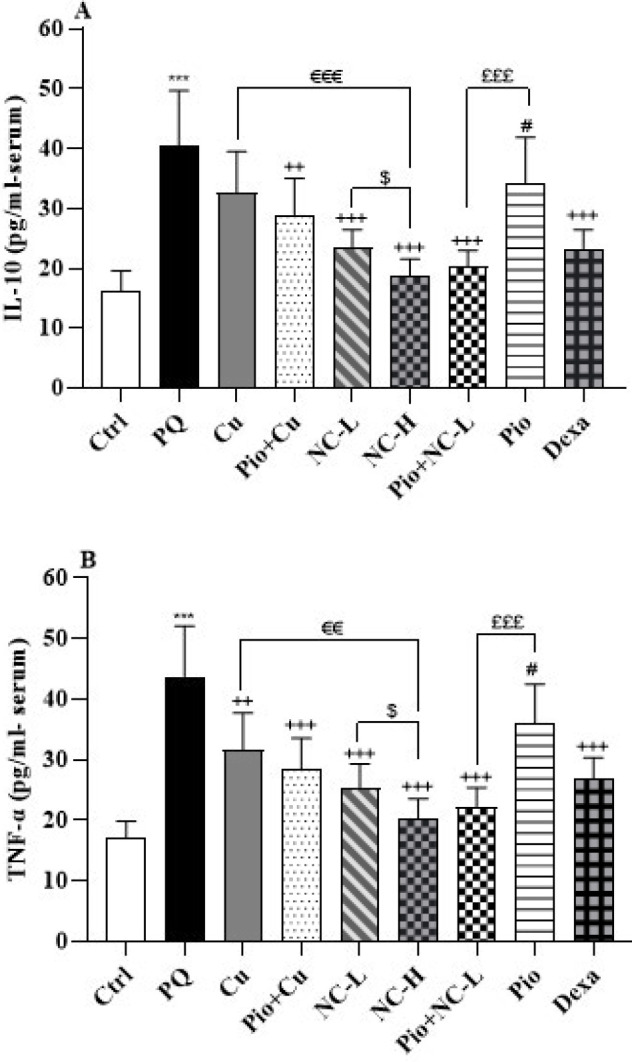
Inflammatory cytokines A: IL-10 and B: TNF-α in the male Wistar rats' serum of the control group (Ctrl), the group exposed to 54 mg/m^3^ paraquat aerosol (PQ), exposing groups to PQ and treated with curcumin (30 mg/kg, 16 days, gavage) (Cu), nanocurcumin (2 mg/kg, 16 days, gavage) (NC-L), nanocurcumin (8 mg/kg, 16 days, gavage) (NC-H), Cu and pioglitazone (5 mg/kg, 16 days, IP) (Pio+ Cu), Pio+ NC-L, Pio, and dexamethasone (0.03 mg/kg, 16 days, IP) (Dexa)

PQ exposure resulted in increased levels of IL-10 and TNF-α compared to the rats in the control group (*P*<0.001 for both) (Figure 5).

Pio + Cu (*P*<0.01), NC-L, NC-H, Pio + NC-L, and Dexa (*P*<0.001 for all) groups showed declined amounts of IL-10 (Figure 5A). Additionally, Cu (*P*<0.01), Pio+ Cu, NC-L, NC-H, Pio+ NC-L, and Dexa (*P*<0.001 for all) groups showed reduced TNF-α levels (Figure 5B).

In the NC-H group, there were significantly decreased levels of IL-10 and TNF-α compared to the NC-L group (*P*<0.05 for both).The administration of NC-H could lessen IL-10 (*P*<0.001) and TNF-α (*P*<0.01) levels more than the supplementation of Cu. 

Receiving Pio + NC-L attenuated IL-10 and TNF-α levels more than the supplementation of Pio (*P*<0.001).

Dexa group showed lessened IL-10 and TNF-α levels more than the Pio group (*P*<0.05).

## Discussion

The current study aimed to examine the probable preventive effects of curcumin, nanocurcumin, and pioglitazone against systemic inflammation and oxidative stress induced by inhaled paraquat in rats. The obtained results revealed that inhaling PQ led to an elevation in total WBC, differential WBC, MDA, IL-10, and TNF-α levels while causing a decrease in blood levels of CAT, SOD, and thiol. However, the treatment groups (Cu, NC-L, NC-H, Pio+ Cu, Pio+ NC-L, Pio, and Dexa) had improved changes caused by PQ. Moreover, the Pio + Cu or NC-L-treated groups exhibited greater improvements in most parameters than the effects observed when using the three substances individually.

Previous studies have demonstrated that PQ induces systemic inflammation and oxidative stress. This is evident from increased macrophages levels of IL-6 and TNF-α (37). Additionally, PQ causes a rise in the number of white blood cells, both total and differential, which is indicative of systemic inflammation. Systemic oxidative stress was also indicated, as seen by higher MDA and decreased thiol content, as well as decreased CAT and SOD activities (6, 8). Animal studies have demonstrated that inhaling PQ increases blood MDA levels while decreasing anti-oxidant levels such as CAT, SOD, and thiol (31). These findings reinforce the findings of the current investigation, which used inhaled PQ delivery to simulate the exposure encountered by farmers who use this herbicide.

Cu, NC-L, NC-H, Pio, and Dexa treatments had similar effects on PQ-exposed rats. Each treatment lowered total and differential white blood cell counts and decreased amounts of oxidants such as MDA. They also resulted in increased anti-oxidant levels, particularly thiol, along with increased SOD and CAT activities. Furthermore, the treatments attenuated the levels of inflammatory cytokines, including TNF-α and IL-10. The data showed that Cl, Cu, Pio, and Dexa treatments positively impacted systemic oxidative stress and inflammation in rats triggered by inhaled PQ. Notably, Dexa, as an anti-inflammatory supplement, had similar effects to Cu and NC, demonstrating that Cu and NC had anti-inflammatory properties in PQ-induced systemic oxidative stress and inflammation. In line with our results, in a laboratory asthma model, Cu was observed to improve inflammatory mediators, WBC count, and total and differential count of bronchoalveolar lavage fluid (38). Likewise, in a recent investigation, the supplementation of ethanolic extract of *C. longa* and NC as therapeutic agents reduced oxidative stress (enhanced serum levels of SOD, thiol, CAT, and decreased MDA levels) and inflammation (lowered TNF-α and enhanced IL-10) in rats with PQ-induced systemic inflammation and oxidative stress in rats (8). Thus, our findings, along with the results of other studies, demonstrate that Cu, NC, Pio, and Dexa administration have a beneficial effect on systemic inflammation and oxidative stress caused by PQ by preventing the production of inflammatory prostaglandins (39), a rise in the activity of SOD, glutathione peroxidase, and CAT enzymes, as well as immunomodulatory effects (22).

IL-10 plays an important anti-inflammatory cytokine that plays a significant role in inflammatory responses (40). Increased IL-10 levels in the PQ group suggest its involvement in the inflammatory effects of PQ exposure (41). Reduction of IL-10 levels in treatment groups and improved total and differential WBC and oxidative stress markers indicated its potential effect on the inflammatory process induced by inhaled PQ (42). The reduction effects of treated agents, mainly Cu and NC, on IL-10 may suggest that the overall role of IL-10 can vary significantly in the inflammatory process based on the underlying inflammatory inducer, which should be clarified in further studies. Previous studies also indicate increased IL-10 levels in lung injury due to inhaled PQ, which could be attributed to the body’s attempt to counteract the inflammatory processes triggered by PQ aerosol. The reduction of IL-10 in these studies after treatment with *Crocus sativus* and NC indicates their anti-inflammatory properties (2), which support the current study’s findings.

Since NC-H was more effective than NC-L in ameliorating the alterations induced by PQ, it can be concluded that the response of NC is dose-dependent. Moreover, NC revealed more beneficial effects in most factors than Cu. The current study examined the effects of two different doses of NC (2 and 8 mg/kg) but one dose of Cu (30 mg/kg). Our findings are consistent with a previous study that reported similar results (8). It is worth noting that the doses of NC used in this study were significantly lower than Cu. Hence, a significant finding of this study is that NC, even at much lower doses, exhibits more potent effects than Cu.

Previous research has documented the anti-inflammatory and anti-oxidant properties of PPAR-γ receptor agonists, including Pio. In particular, Pio administration at doses of 5 and 10 mg/kg has been found to inhibit the increase in myeloperoxidase activity and the expression of TNF-α protein and messenger ribonucleic acid (mRNA) (8). These investigations confirm the current study’s outcomes on the effects of pioglitazone on oxidative stress induced by inhaled PQ and systemic inflammation.

Another significant observation in this study was the synergistic effects observed when combining Cu and NC-L with Pio were administered. It was observed that Pio + Cu and Pio + NC-L had more profound effects on all investigated variables than did Pio, Cu, and NC-L alone. These data suggest that Cu and NC may affect PPAR-γ receptors. However, more study incorporating the presence of a PPAR-γ receptor antagonist medication is required to verify the impact of these substances on PPAR-γ receptors.

The findings of this study provided novel insights into the effects of Cu and NC on systemic inflammation and oxidative stress induced by inhaled PQ. Importantly, these results suggest the involvement of PPAR-γ in mediating the effects of Cu and NC. This study is the first to demonstrate such effects, expanding our understanding of the potential mechanisms underlying the preventive effects of Cu and NC in this field. Although, further studies should investigate the anti-apoptotic effects of Cu and NC both *in vitro* and *in vivo*. Additionally, exploring the effects of Cu and NC on the inhibition of the nuclear factor erythroid 2-related factor 2 (Nrf2) signaling pathway would provide valuable evidence and enhance our understanding of the functional significance of extracellular vesicles derived from human mesenchymal stem cells (hMSCs) in the treatment of systemic inflammation disorders. Moreover, it is crucial to examine the potential protective effects of Cu and NC in cell models to support the present study’s findings. These future studies would lead to a more comprehensive and reliable understanding of the therapeutic potential of Cu and NC. 

Dexa was used as a positive control group in this investigation since it has been found to have therapeutic effects by exerting anti-oxidant and anti-inflammatory effects in contrast to PQ-induced systemic inflammation and oxidative stress (8). Our results also showed no significant difference between the effects of Dexa and NC-L or NC-H on most measured variables.

According to pharmacology toxicological principles, low doses of agents that do not lead to maximum response should be used to investigate synergistic effects. The maximum response may be achieved in a high dose, but the synergic effect was not shown. Therefore, low doses of NC, Pio, and Cu were used in the current study to evaluate their combination effect and study the synergic effect.

Regarding the evaluation of potential liver and kidney toxicity of NC, a study (43) that administered 2.00 g/kg nanocurcumin for 50 days did not show any impact on liver and kidney histology and biochemical parameters. Additionally, research conducted by Tohamy *et al*. (44) indicated the hepatorenal protective effects of 100 mg/kg for 14 days NC against nano-copper oxide-induced toxicity in rats. These studies provide valuable insights into the safety profile of NC in relation to liver and kidney function.

*** *P*<0.001 statistical differences versus Ctrl group; +++ *P*<0.001 statistical differences versus PQ group; $ P<0.05 statistical differences versus NC-H group; # *P*<0.05, ## *P*<0.01, and ### *P*<0.001 versus Dexa group; € *P*<0.05 and €€€ *P*<0.001versus Cu group; £ *P*<0.05, ££ *P*<0.01, and £££ *P*<0.001 versus Pio-Cu and Pio-NC-L. The results are expressed as mean ± SEM (n= 7 in each group). One-way ANOVA followed by Tukey's multiple comparison test was applied to compare different groups.

*** *P*<0.001 statistical differences versus Ctrl group; + *P*<0.05 and +++ *P*<0.001 statistical differences versus PQ group; $ *P*<0.05 statistical differences versus NC-H group; ### *P*<0.001 versus Dexa group; € *P*<0.05 and €€€ *P*<0.001versus Cu group; £ *P*<0.05, ££ *P*<0.01, and £££ *P*<0.001 versus Pio-Cu and Pio-NC-L. The results are expressed as mean ± SEM (n= 7 in each group). One-way ANOVA followed by Tukey's multiple comparison test was applied to compare different groups.

*** *P*<0.001 statistical differences versus Ctrl group; + *P*<0.05, ++ *P*<0.01, and +++ *P*<0.001 statistical differences versus PQ group; $ *P*<0.05 and $$ *P*<0.001 statistical differences versus NC-H group; # *P*<0.05 and ## *P*<0.01 versus Dexa group; €€ *P*<0.01 and €€€ P<0.001 versus Cu group; ££ *P*<0.01 and £££ *P*<0.001 versus Pio-Cu and Pio-NC-L. The results are expressed as mean ± SEM (n= 7 in each group). One-way ANOVA followed by Tukey’s multiple comparison test was applied to compare different groups.

*** *P*<0.001 statistical differences versus Ctrl group; ++ *P*<0.01 and +++ *P*<0.001 statistical differences versus PQ group; $ P<0.05 statistical differences versus NC-H group; # *P*<0.05 versus Dexa group; €€ *P*<0.01 and €€€ *P*<0.001versus Cu group; £££ *P*<0.001 versus Pio-Cu and Pio-NC-L. The results are expressed as mean ± SEM (n= 7 in each group). One-way ANOVA followed by Tukey’s multiple comparisons test was applied to compare different groups.

## Conclusion

Treatment with Cu, NC, Pio, or Dexa effectively mitigates the PQ-induced systemic inflammation and oxidative stress. Interestingly, the combination of Pio with Cu or NC displays more remarkable amelioration in most parameters compared to individual treatment. These results propose a synergistic effect between Pio and Cu/NC, suggesting that the effects of Cu are possibly mediated through PPARγ mechanisms.

Our findings generally highlight the potential of Cu, NC, and Pio in alleviating PQ-induced systemic inflammation and oxidative stress. Further investigations are necessary to elucidate the underlying mechanisms and explore these substances’ therapeutic potential in managing related disorders.

## Data Availability

The datasets used and/or analyzed during the current study are available from the corresponding author upon reasonable request.

## Declaration

During the preparation of this work, the author(s) used ChatGPT and Quillbot to rephrase to reduce plagiarism and improve the language and grammar. After using this tool/ service, the author(s) reviewed and edited the content as needed and take(s) full responsibility for the publication’s content.
